# Zusammenarbeit von Intensivmedizin und Palliativmedizin: Eine Bestandsaufnahme an den deutschen onkologischen Spitzenzentren

**DOI:** 10.1007/s00063-020-00712-0

**Published:** 2020-08-07

**Authors:** J. Berendt, C. Ostgathe, S. T. Simon, M. Tewes, D. Schlieper, M. Schallenburger, S. Meier, S. Gahr, J. Schwartz, M. Neukirchen

**Affiliations:** 1 Palliativmedizinische Abteilung, Universitätsklinikum Erlangen, Comprehensive Cancer Center EMN-Erlangen, Friedrich-Alexander-Universität Erlangen-Nürnberg (FAU), Erlangen, Deutschland; 2 Zentrum für Palliativmedizin, Centrum für Integrierte Onkologie Aachen, Bonn, Köln, Düsseldorf, Uniklinik Köln, Köln, Deutschland; 3 Westdeutsches Tumorzentrum, Innere Klinik (Tumorforschung), Universitätsklinikum Essen, Essen, Deutschland; 4 Interdisziplinäres Zentrum für Palliativmedizin, Universitätsklinikum, Heinrich-Heine-Universität Düsseldorf, Moorenstraße 5, 40225 Düsseldorf, Deutschland; 5 Klinik für Anästhesiologie, Universitätsklinikum, Heinrich-Heine-Universität Düsseldorf, Düsseldorf, Deutschland

**Keywords:** Triggerfaktoren, Intensivpatient, Therapiezielfindung, Palliativdienst, Interdisziplinäre Zusammenarbeit, Trigger factors, Intensive care patient, Therapeutic goal setting, Palliative care consultation service, Interdisciplinary collaboration

## Abstract

**Hintergrund:**

Die interdisziplinäre Zusammenarbeit zwischen Intensivmedizin und Palliativmedizin kann die Versorgungsqualität verbessern. Das Ausmaß dieser Zusammenarbeit ist aber bisher kaum untersucht.

**Ziel der Arbeit:**

Es sollten die angebotenen und in Anspruch genommenen palliativmedizinischen Unterstützungsangebote auf den Intensivstationen deutscher onkologischer Spitzenzentren erfasst werden.

**Material und Methoden:**

Durchgeführt wurde eine quantitativ-qualitative, deskriptive Umfrage an den 16 von der Stiftung Deutsche Krebshilfe geförderten Zentren. Die im quantitativen Teil erfragten Häufigkeiten werden als Mittelwert und Median mit den jeweiligen Streumaßen dargestellt, während die im qualitativen Teil erhobenen Triggerfaktoren mit einer Inhaltsanalyse nach Mayring ausgewertet wurden.

**Ergebnisse:**

Von Juli bis August 2017 konnten Angaben aus 15 von 16 onkologischen Spitzenzentren (94 %) erfasst werden. Im Jahr 2016 wurden im Median 33 Intensivpatienten (Min. 0, Max. 100) palliativmedizinisch vorgestellt und 9 Patienten (Min. 1, Max. 30) auf eine Palliativstation verlegt. Regelmäßige intensivmedizinisch-palliativmedizinische Visiten sowie ein Screening-Tool zur Einbindung der spezialisierten Palliativmedizin sind an zwei onkologischen Spitzenzentren implementiert. Anhand von 23 genannten Triggern, die auf der Intensivstation eine palliativmedizinische Mitbehandlung ausgelöst haben, lassen sich nach qualitativer Analyse die drei Kategorien „Entscheidung und Einstellung des Teams“, „Zustand des Patienten“ und „Wunsch von Patienten und Angehörigen“ ableiten.

**Diskussion:**

Trotz eines verfügbaren Angebots werden palliativmedizinische Ressourcen in den intensivmedizinischen Abteilungen der onkologischen Spitzenzentren immer noch selten genutzt. In die tägliche Routine integrierte Angebote wie Screening-Tools oder gemeinsame Visiten könnten die Ausnutzung der angebotenen palliativmedizinischen Ressourcen erhöhen und die Versorgungsqualität verbessern.

## Abstract (en)

**Background:**

For intensive care patients with limited life expectancy the integration of palliative care in intensive care may be beneficial. However, little is known about the extent of this interdisciplinary collaboration.

**Objectives:**

The support given by palliative medicine in German oncological centers and used by the intensive care units should be recorded.

**Material and methods:**

A descriptive survey was conducted in all of the 16 Comprehensive Cancer Centers (CCC) funded by German Cancer Aid. The questionnaires were sent to the head of department of the CCCsʼ specialized palliative care teams. Data were collected for the year 2016. Quantitative data were analysed to establish frequencies, given as mean and median. A qualitative section asked for trigger factors, i.e., patient characteristics triggering a palliative care consultation. Evaluation was inductively carried out by content analysis according to Mayring.

**Results:**

Data from 15 of the 16 CCCs (94%) were obtained between July and August 2017. In 2016, the median of intensive care patients with palliative care consultations was 33 (minimum 0, maximum 100). The median of nine patients were transferred from an intensive care unit to a palliative care unit (minimum 1, maximum 30). Multidisciplinary ward rounds by both intensive and palliative care staff were available in two CCCs on a regular basis. Two CCCs implemented screening tools to integrate specialized palliative care into intensive care. From 23 responses concerning triggers, three categories were established, i.e., “team’s decision and attitude”, “patient’s condition” and “desires of patients and relatives”.

**Conclusions:**

Palliative care is available in German CCCs. However, the degree of integration of specialized palliative care into intensive care units is low. Screening tools are available to identify patients with complex needs and to trigger a palliative care consultation. These tools, as well as joint ward rounds of intensive and palliative care staff, can improve the quality of patient centred care.

Die patientenorientierte Behandlung von Intensivpatienten hat weit mehr zum Ziel als die reine Lebenszeitmaximierung. Für die bestmögliche Versorgungsqualität und Lebensqualität können die Patienten von einer engen Zusammenarbeit zwischen der Intensivstation und der Palliativmedizin profitieren [18–20]. Doch wie gestaltet sich bislang der Brückenschlag zwischen der Intensivmedizin und Palliativmedizin? Eine Bestandsaufnahme bei den von der deutschen Krebshilfe geförderten onkologischen Spitzenzentren (Comprehensive Cancer Center [CCC]) soll einen ersten Eindruck dazu vermitteln.

## Hintergrund

Sowohl in der Intensivmedizin als auch in der Palliativmedizin werden Patienten behandelt, die an lebenslimitierenden Erkrankungen leiden. Es ist daher naheliegend, dass es Intensivpatienten gibt, die von einer spezialisierten palliativmedizinischen Mitbehandlung durch einen Palliativdienst oder sogar von einer palliativmedizinischen stationären Behandlung profitieren. Hierzu ist eine frühzeitige Integration palliativer Prinzipien in die Intensivmedizin möglich [19, 22]. Die S3-Leitlinie „Palliativmedizin für Patienten mit einer nicht heilbaren Krebserkrankung“ empfiehlt generell eine frühzeitige Integration („early integration“) bei Tumorpatienten. Patienten sowie deren Vorsorgebevollmächtigte, Betreuer und Angehörige sollen bereits bei Diagnosestellung einer lebenslimitierenden Erkrankung über die Möglichkeiten der Palliativversorgung informiert werden. Außerdem soll ihnen während des stationären Aufenthalts der Kontakt mit einem Palliativdienst angeboten werden [16]. Auch bei Patienten mit lebenslimitierenden nichtonkologischen Grunderkrankungen ist ein früh einsetzendes Behandlungskonzept sinnvoll, damit die Palliativmedizin die krankheitsspezifische Therapie bzw. Begleitung mit besonderem Fokus auf die verbleibende Lebenszeit in einem nichtklinischen Setting und zum Teil mit spezialisierter palliativmedizinischer Ausrichtung aufgrund bestmöglicher Lebensqualität unterstützen kann [3, 19, 20].

Diese Empfehlung gilt ebenso für Patienten, die intensivmedizinisch behandelt werden. In der Literatur sind vielfältige Kriterien für eine Bitte um Mitbehandlung seitens des intensivmedizinischen Teams zu finden [1]. Jedoch werden nicht alle Kriterien von den Intensivärzten akzeptiert und angewendet [2]. Die konsequente Einbindung der Krankenpflege kann helfen, Patienten mit Bedarf an Palliativmedizin zu identifizieren, ohne dass starre Kriterien vorgegeben werden [11, 13].

In deutschen Krankenhäusern sind es überwiegend die Kliniken für Anästhesiologie [6] und Innere Medizin, die zu einem erheblichen Teil sowohl zur Intensivversorgung als auch zur Palliativversorgung beitragen. Obwohl bereits einige Krankenhäuser einen palliativmedizinischen Dienst etabliert haben, fehlt es häufig an weiteren Versorgungsstrukturen und konkreten Maßnahmen der Integration von Palliativmedizin. Hinzu kommt im klinischen Alltag eine heterogene Akzeptanz und Nutzung palliativmedizinsicher Angebote sowohl von Patienten als auch aufseiten der Primärbehandler.

Die Stiftung Deutsche Krebshilfe fördert in Deutschland onkologische Spitzenzentren (Comprehensive Cancer Center [CCC]), um damit wegweisende Strukturen für die Krebsversorgung zu schaffen. Dabei wird eine Behandlung nach einheitlichen, hohen Qualitätsstandards angestrebt. Eine Bestandserhebung unter den CCC ist daher in der Lage, erste Empfehlungen abzuleiten, wie Versorgungsstrukturen im Schnittstellenbereich zwischen Intensiv- und Palliativmedizin idealerweise aussehen sollen. Darüber hinaus kann eine solche Erhebung Hinweise auf einen Verbesserungsbedarf geben. Daher soll in dieser Arbeit die Integration der spezialisierten Palliativmedizin in die Intensivmedizin an den deutschen CCC untersucht werden. Die Fragestellungen lauten: Wie ist die Palliativmedizin aus Sicht der leitenden ärztlichen Vertreter der spezialisierten Palliativmedizin bislang in die Intensivmedizin ihrer jeweiligen Einrichtung integriert? Wie viele Patienten erhalten eine palliativmedizinische Mitbehandlung oder werden direkt von Intensivstationen auf Palliativstationen übernommen? Welche Trigger führen zu einer palliativmedizinischen Mitbehandlung?

## Studiendesign und Untersuchungsmethode

Es handelt sich um ein deskriptives Studiendesign mit quantitativen und qualitativen Daten. Für die Vollerhebung wurde ein Fragebogen entwickelt, der neben der hier dargestellten Zusammenarbeit zwischen Intensivmedizin und Palliativmedizin auch die Implementierung einer spezialisierten ambulanten palliativmedizinischen Sprechstunde, ärztlichen Rotation und von Palliativdiensten erfragte [4, 9]. Der Fragebogen wurde an alle ärztlichen Leitungen der spezialisierten Palliativmedizin in den 16 von der Stiftung Deutsche Krebshilfe geförderten deutschen CCC verschickt. Die Befragten erhielten den Fragebogen im Juli 2017 und konnten ihn bis August 2017 per Fax beantworten. Die Fragen bezogen sich auf das Jahr 2016 [4, 9]. Die Fragen zur Einbindung der spezialisierten Palliativmedizin in die jeweilige Intensivstation (ICU) bzw. Überwachungsstation beinhalteten offene und geschlossene Fragen. Der Fragebogen wurde zuvor in drei CCC erprobt und nach Rückmeldung angepasst. Es wurden Häufigkeitsverteilungen, Mittelwerte, Median und die jeweiligen Streumaße („standard deviation“ [SD]) ermittelt. Die Auswertungen der vorgegebenen Antwortoptionen und die Erfassung der Freitextangaben erfolgten in SPSS (IBM, Armonk, NY, USA). Die Freitextangaben wurden anschließend von zwei wissenschaftlichen Mitarbeitern unabhängig voneinander analysiert und kategorisiert. Dabei wurde die zusammenfassende Inhaltsanalyse nach Mayring angewendet, um induktiv Kategorien zu bilden, die miteinander abgeglichen und konsentiert wurden [21, 25]. Für die induktive Vorgehensweise spricht, dass bisher die Gründe für die Einbindung der spezialisierten Palliativmedizin in die intensivmedizinische Versorgung (Trigger) in der Literatur uneinheitlich definiert sind [1].

## Ergebnisse

Von 16 kontaktierten CCC nahmen 15 (94 %) teil. In den 15 CCC bestanden 6 palliativmedizinische Einheiten seit über 10 Jahren und 7 palliativmedizinische Einheiten seit 5 bis 10 Jahren. In einem CCC lag das Gründungsjahr der spezialisierten Palliativmedizin 3 bis 4 Jahre zurück, während ein CCC noch keine palliativmedizinische Abteilung hatte. Elf der 15 Zentren (73 %) boten einen palliativmedizinischen Dienst an.

Der Palliativdienst wurde im Median bei 33 Intensivpatienten pro Jahr (Spanne: 0–100) eingebunden. Für die direkten Übernahmen von Patienten der Intensivstation auf die Palliativstation ergab sich im CCC-Netzwerk ein Median von 9 Übernahmen pro Jahr (Spanne: 1–30). Die berichteten Zahlen sind in Tab. 1 aufgeführt.

**Table 1:** 

CCC Nr.	Einbindung in Palliativdienst (Anzahl der Patienten)	Übernahmen von ICU auf Palliativstation (Anzahl der Patienten)	Palliativdienst verfügbar
1	100	30	Ja
2	6	6	Ja
3	40	5	Ja
4	52	22	Ja
5	0	Keine Angabe	Nein
6	0	4	Nein
7	25	10	Ja
8	0	Keine Angabe	Nein
9	0	2	Nein
10	5	20	Ja
11	0	1	Ja
12	25	20	Ja
13	33	7	Ja
14	60	Keine Angabe	Ja
15	100	20	Ja

In 2 der 15 CCC (13 %) wurden bisher regelmäßige intensivmedizinisch-palliativmedizinische Visiten durchgeführt. Ein drittes CCC gab an, intensivmedizinisch-palliativmedizinische Visiten nicht regelmäßig, aber bei Bedarf durchzuführen.

In ebenfalls 2 der 15 CCC (13 %) ist auf den Intensivstationen ein Screening-Tool für die Einbindung der spezialisierten Palliativmedizin etabliert.

Insgesamt benannten 11 Befragte Triggerfaktoren für ein Hinzuziehen des palliativmedizinischen Teams. Hierbei konnten 23 Triggerfaktoren erfasst werden, die in der täglichen Praxis genutzt werden. Diese lassen sich in drei Kategorien zusammenfassen: „Entscheidung und Einstellung des Teams“, „Zustand des Patienten“ und „Wunsch von Patienten und Angehörigen“ (Tab. 2).

**Table 2:** 

Häufigkeit der Nennungen	Genannter Trigger (Freitext)	Generalisierung (Häufigkeit der Nennungen)	Kategorie (Häufigkeit der Nennungen)
3	Therapiezielklärung, -begrenzung, -änderung	Therapiezieländerung (4)	Entscheidung und Einstellung des Teams (13)
1	Therapieeskalation erfolgt durch die Kollegen direkt in Absprache mit den Angehörigen		
1	Persönlich-individuelle Entscheidung, Einstellung der Ärzte (mitarbeitergetriggert)	Entscheidung und Einstellung der Mitarbeiter (2)	
1	Einstellung der Ärzte		
1	Bedarf für palliativmedizinischen Sozialdienst	Unklare Versorgung (2)	
1	Unklare Weiterversorgung		
1	Vorgespräch mit Patienten/Angehörigen zur geplanten palliativmedizinischen Versorgung	Vorgespräch zur palliativmedizinischen Versorgung (1)	
1	Visite des Oberarzthintergrunddiensts der Klinik […^a^] (u. a. Oberärzte mit palliativmedizinischer Weiterbildung)	Palliativmedizinische Zusatzweiterbildung (2)	
1	Sehr komplexe Verhältnisse […^a^], in einigen Stationen^a^ sind qualifizierte Palliativmediziner mit Zusatzweiterbildung verantwortlich tätig		
1	Ggf. persönliche Bekanntheit	Persönlicher Kontakt (2)	
1	Persönliche Ansprache		
2	Symptome (symptomatisch und/oder psychologisch)	Symptome (2)	Zustand des Patienten (6)
1	Nicht heilbare Grunderkrankung	Nicht heilbare Grunderkrankung (1)	
1	Sterbephase	Sterbephase (1)	
1	Belastetes Familiensystem	Belastete Situation (2)	
1	Schwierige ethische Situation		
2	Angehörigenwunsch	Übernahmewunsch von Patienten und Angehörigen (4)	Wunsch von Patienten und Angehörigen (4)
1	Patientenwunsch		
1	Übernahmewunsch Palliativstation		

^a^Diese Detailinformation wurde zur Anonymisierung der Antworten gelöscht oder nur sinngemäß wiedergegeben

## Diskussion

Die Zusammenarbeit von Palliativmedizin und Intensivmedizin in den deutschen onkologischen Spitzenzentren ist sehr unterschiedlich ausgestaltet. Ursächlich hierfür sind zunächst strukturelle Unterschiede des palliativmedizinischen Angebots. Lücken in der Verfügbarkeit spezialisierter palliativmedizinischer Angebote können Auswirkungen auf die Arbeit der Teams von Intensivstationen haben, wie national und international festgehalten wird [18, 24]. Während nahezu alle Standorte Palliativstationen vorhielten (und vorhalten), boten in 2016 nur ca. 70 % der Zentren einen palliativmedizinischen Dienst an. Diese beratenden Dienste als ein Modell der Palliativversorgung für die Intensivpflege [18] stellen ein elementares Bindeglied zwischen stationär palliativ- und intensivmedizinischer Versorgung dar und sollten daher ausgebaut werden. Sowohl die S3-Leitlinie „Palliativmedizin für Patienten mit einer nicht heilbaren Krebserkrankung“ [16] als auch die Best-Practice-Empfehlung der Stiftung Deutsche Krebshilfe [27] sieht in jedem CCC sowohl eine Palliativstation als auch einen palliativmedizinischen Dienst vor. Durch das Zusatzentgelt ZE 2018-133 sind hier erst in den letzten Jahren neue potenzielle Finanzierungsmöglichkeiten geschaffen worden, sodass, wenn das Zusatzentgelt kalkuliert sein wird, mit einer besseren Versorgung zu rechnen ist. Nach erfolgreicher Etablierung der strukturellen Voraussetzungen für eine spezialisierte palliativmedizinische Behandlung an den CCC stellen diese eine Vorbildfunktion für weitere Versorger dar. Obwohl in vielen CCC Strukturen zur palliativmedizinischen Mitbehandlung zur Verfügung stehen [5] und die Inanspruchnahme des Palliativdiensts von anderen Abteilungen regelhaft erfolgt [7, 10], ist die Anzahl der Anfragen der Intensivmedizin an die Palliativmedizin in den meisten CCC bisher noch verhältnismäßig gering, zum Beispiel unter Einbezug der Ergebnisse einer österreichischen Studie [19]. Die vorliegende Arbeit zeigt auf, dass es nicht grundsätzlich an einer Lücke in der generellen Verfügbarkeit eines Palliativdiensts liegt und demnach auch Modelle zur Integration von Palliativmedizin in die Intensivmedizin [18, 19, 28] in die Anwendung kommen sollten. Regelmäßige (z. B. zweiwöchentliche) intensivmedizinisch-palliativmedizinische Visiten, die sich zu einem unmittelbaren Booster palliativmedizinischer Aktivität auf einer Intensivstation entwickeln können [19], sowie standardisierte Screening-Tools werden auch an den Zentren mit existierenden Palliativdiensten nur unregelmäßig angeboten.

Im Alltag werden Intensivmediziner im Schnitt einmal pro Woche mit Therapiezieländerungen konfrontiert, in deren Rahmen eine zunächst kurativ intendierte Behandlung in eine palliative Behandlung überführt wird [14]. 35 % der Ärzte fühlen sich bei dieser Entscheidung unsicher [14]. Insbesondere deshalb muss sichergestellt sein, dass zum einen genügend Intensivmediziner mit palliativmedizinischem Basiswissen zur Verfügung stehen und dass zum anderen im Falle hoher Symptomlast die zur Verfügung stehenden Strukturen der spezialisierten Palliativmedizin bekannt sind [29, 30]. Außerdem sollte ein standardisiertes Vorgehen zur Anforderung des Palliativdiensts etabliert sein [15]. Bisher konnte diesbezüglich jedoch noch unzureichendes Wissen festgestellt werden [15, 29, 30]. Die ärztliche und pflegerische palliativmedizinische Aus‑, Fort- und Weiterbildung in den CCC bedarf der Weiterentwicklung [14]. Kommunikative Fähigkeiten zur Übermittlung schlechter Nachrichten am Lebensende sind verbesserungsfähig [31]. Auch wenn sich viele Patienten ihres unmittelbar bevorstehenden Lebensendes bewusst sind, hat nur ein geringerer Anteil der Patienten diesen Punkt mit einem Arzt besprochen [12]. Jedoch werden Therapiezieländerungen zunehmend standardisiert dokumentiert, z. B. in Anlehnung an den Dokumentationsbogen der Sektion Ethik der Deutschen Interdisziplinären Vereinigung für Intensiv- und Notfallmedizin [13, 17, 23, 26]. Patienten und deren Vorsorgebevollmächtigte befürworten zu Beginn eines protrahierten postoperativen Verlaufs häufig Maximaltherapie, was sich im Krankheitsverlauf jedoch ändern kann [12]. Eine derartige Meinungsänderung ist oft mit dem Wunsch nach einer Therapiezieländerung verknüpft und betrifft insbesondere Patienten, die durch Langzeitintensivtherapie hoch belastet sind, die einen schlechten funktionellen Status aufweisen oder die nach langer Intensivbehandlung unter reduzierter Lebensqualität leiden. Ein Palliativdienst kann dann im Laufe der Behandlung für Patienten, Angehörige und die behandelnden Berufsgruppen ergänzend unterstützen. Mit dem Behandlungsfokus auf Symptomkontrolle und Lebensqualität kann der Patient bis zum Lebensende auf der Intensiv- oder Normalstation verbleiben. Ein gewisser Prozentsatz der Intensivpatienten bedarf nach palliativmedizinischer Mitbehandlung aber auch einer Übernahme auf die Palliativstation [8]. Der Anteil der von einem Palliativdienst mitbehandelten Patienten, die in der Folge auf die Palliativstation übernommen wurden, liegt in unserer Untersuchung bei 27 %. Dass nicht jeder mitbehandelte Patient, selbst nach Therapiezieländerung auf ein palliatives Therapieziel, auf die Palliativstation übernommen werden kann, ist unter anderem darauf zurückzuführen, dass Therapiezieländerungen auf Intensivstationen häufig mit der Einstellung von Organersatzverfahren einhergehen (z. B. Katecholamintherapie bei Herzinsuffizienz oder Sepsis, Beatmung bei respiratorischer Insuffizienz oder kontinuierliche Nierenersatztherapie bei akutem Nierenversagen). Zumeist treten diese Patienten dann sehr schnell in die Sterbephase ein, in der eine Verlegung, verbunden mit einem belastenden Umgebungswechsel, nicht mehr indiziert bzw. vertretbar ist. Außerdem führt nicht jede Therapiezieländerung zur Einstellung sämtlicher intensivmedizinischer Maßnahmen, sodass auch dann eine Verlegung in die spezialisierte Palliativmedizin nicht möglich ist [18].

Screening-Tools und die darin genannten standardisierten Triggerfaktoren könnten die Häufigkeit palliativmedizinischer Mitbehandlungen auch auf Intensivstationen erhöhen, doch unter Intensivmedizinern besteht nur eine geringe Akzeptanz bisher publizierter Faktoren [2]. Die im Rahmen der qualitativen Inhaltsanalyse erfassten Trigger decken sich nahezu vollständig mit denen in der bisher veröffentlichten Literatur [1]. Lediglich die Gruppe der Triggerfaktoren mit dem Schwerpunkt „Entscheidung und Einstellung des Teams“ wird von den befragten leitenden Ärzten der Palliativmedizin als zusätzlicher Trigger genannt. Sowohl bei den befragten Palliativmedizinern als auch bei jungen Intensivmedizinern scheinen die beiden Aspekte „Wunsch von Patienten und Angehörigen“ und „Zustand des Patienten“ die akzeptiertesten Auslöser für eine palliativmedizinische Mitbehandlung zu sein [2]. Diesbezüglich wären weitere Studienergebnisse bezogen auf die Sinnhaftigkeit einzelner Triggerfaktoren für eine palliativmedizinische Mitbehandlung von Intensivpatienten wünschenswert, um dann evidenzbasiert Screening-Tools zu entwickeln. Regelmäßige intensivmedizinisch-palliativmedizinische Visiten könnten zu einer Optimierung (und damit Vereinheitlichung) der Behandlung beitragen. Im Rahmen dieser Visite werden in einem gemeinsamen Entscheidungsprozess von primärbehandelnden Intensivmedizinern und Palliativmedizinern Intensivpatienten identifiziert, die von einer multiprofessionellen palliativmedizinischen Mitbehandlung profitieren können. Solche gemeinsamen Visiten werden aktuell nur in 2 der CCC regelmäßig durchgeführt.

Die Ergebnisse zeigen, dass die Etablierung eines palliativmedizinischen Diensts in einem CCC (und damit allgemein in jedem größeren Krankenhaus) möglich ist. Das Vorhandensein eines palliativmedizinischen Diensts stellt eine wichtige Grundvoraussetzung für die fachübergreifende Zusammenarbeit zwischen der Intensiv- und Palliativmedizin dar. Screening-Tools und gemeinsame Visiten von Intensivmedizinern und Palliativmedizinern helfen dabei, mögliche Palliativpatienten auf Intensivstationen frühzeitig zu erkennen [19]. Diese hilfreichen Tools sind bisher allerdings auch in den CCC nur selten etabliert, sodass eine weitere Verbreitung zum Beispiel im Rahmen zukünftiger Zertifizierungen vorgeschlagen werden könnte.

### Limitationen

Die Studie basiert auf einer Befragung einer hochselektionierten Kohorte. Dennoch liegt den Ergebnissen bei Antworten aus 15 von 16 geförderten CCC eine Vollerhebung zugrunde. Befragt wurden die leitenden Ärzte der spezialisierten Palliativmedizin. Intensivmediziner könnten den Bedarf anders einschätzen und vor allem die Trigger für eine Anfrage oder für einen Übernahmewunsch anders werten. Gründe für eine Nichtmitbehandlung durch ein spezialisiertes palliativmedizinisches Team werden nicht erfasst. Unbeleuchtet bleibt dadurch, inwiefern ein Bewusstsein des palliativmedizinischen Angebots und ein Abwägen seitens des intensivmedizinischen Teams, bevor Konsilanfragen an den Palliativdienst ausgeschlossen oder gestellt werden, tragend sind. Hierzu empfiehlt sich eine eigenständige qualitative und/oder quantitative Erhebung. Eine weitere Limitation der Ergebnisse besteht darin, dass Schätzwerte für die Angaben zur Übernahmerate bzw. zu den getätigten Mitbehandlungen berücksichtigt wurden, da die Freitextangaben teilweise mit „ca.“ oder „~“ deklariert waren oder eine Spanne angegeben wurde. Im Falle einer Spanne wurde bei der Auswertung der Maximalwert hinzugezogen. Die Problematik angegebener Spannwerte ist auf die Papierversion des Fragebogens zurückzuführen. Ein Online-Fragebogen mit vordefinierten Feldern hätte eine Angabe von Spannwerten verhindert. Bei der Papierversion des Fragebogens hätte sich der schriftliche Hinweis empfohlen, dass lediglich nur eine Zahl anzugeben ist. Die Gesamtzahl der intensivmedizinisch behandelten Patienten bleibt unberücksichtigt, sodass die erhobenen Zahlen nicht ins Verhältnis gesetzt werden konnten. Zuweisungszahlen anderer Abteilungen wurden nicht erhoben und verglichen. Bei der Abfrage wurde nicht zwischen onkologischen und nichtonkologischen Patienten unterschieden, da die Zahlen zu klein ausgefallen wären. Die Bestandserhebung gibt schlaglichtartig den Bestand des Jahres 2016 wieder. Eine erneute, erweiterte Umfrage ist wünschenswert, um eine zeitliche Entwicklung ableiten zu können.

## Fazit für die Praxis

- Der Palliativdienst stellt für die Versorgung von Intensivpatienten neben der Palliativstation ein Kernelement der spezialisierten palliativmedizinischen Versorgung dar.
- Die Gründe für eine palliativmedizinische Mitbehandlung von Intensivpatienten lassen sich einordnen in die Kategorien „Entscheidung und Einstellung des Teams“, „Zustand des Patienten“ und „Wunsch von Patienten und Angehörigen“.
- Regelmäßige intensivmedizinisch-palliativmedizinische Visiten und standardisierte Screening-Tools können helfen, Intensivpatienten mit palliativmedizinischem Bedarf zu identifizieren.
- Ein nicht unerheblicher Anteil der Intensivpatienten bedarf nach palliativmedizinischer Mitbehandlung einer Übernahme auf die Palliativstation.
